# Metabolic vulnerability of cancer stem cells and their niche

**DOI:** 10.3389/fphar.2024.1375993

**Published:** 2024-04-10

**Authors:** Laura Marrone, Simona Romano, Chiara Malasomma, Valeria Di Giacomo, Andrea Cerullo, Rosetta Abate, Marialuisa Alessandra Vecchione, Deborah Fratantonio, Maria Fiammetta Romano

**Affiliations:** ^1^ Department of Molecular Medicine and Medical Biotechnology, University of Naples Federico II, Naples, Italy; ^2^ Department of Medicine and Surgery, LUM University Giuseppe Degennaro, Bari, Italy

**Keywords:** cancer stem cells, tumor dormancy, tumor associated macrophages, oxidative metabolism, anti-mitochondrial drugs in clinical trials

## Abstract

Cancer stem cells (CSC) are the leading cause of the failure of anti-tumor treatments. These aggressive cancer cells are preserved and sustained by adjacent cells forming a specialized microenvironment, termed niche, among which tumor-associated macrophages (TAMs) are critical players. The cycle of tricarboxylic acids, fatty acid oxidation path, and electron transport chain have been proven to play central roles in the development and maintenance of CSCs and TAMs. By improving their oxidative metabolism, cancer cells are able to extract more energy from nutrients, which allows them to survive in nutritionally defective environments. Because mitochondria are crucial bioenergetic hubs and sites of these metabolic pathways, major hopes are posed for drugs targeting mitochondria. A wide range of medications targeting mitochondria, electron transport chain complexes, or oxidative enzymes are currently investigated in phase 1 and phase 2 clinical trials against hard-to-treat tumors. This review article aims to highlight recent literature on the metabolic adaptations of CSCs and their supporting macrophages. A focus is provided on the resistance and dormancy behaviors that give CSCs a selection advantage and quiescence capacity in particularly hostile microenvironments and the role of TAMs in supporting these attitudes. The article also describes medicaments that have demonstrated a robust ability to disrupt core oxidative metabolism in preclinical cancer studies and are currently being tested in clinical trials.

## Introduction

There is a consensus that conventional cancer treatments fail due to the failure to eliminate tumor stem cells (CSCs), i.e., the stem, regenerative, and undifferentiated component of the tumor. Tumor cells that survive treatment are more difficult to eradicate, are aggressive, are responsible for relapses, and possess stem-like properties overall (Baccelli and Trump, 2012; Fan et al., 2023). The expression of surface stemness markers (e.g., CD44, CD133, CD25, ABC transporters), stemness genes (e.g., OCT4, SOX2, NANOG) and aldehyde dehydrogenase 1-ADH1; the capacity for tumorigenicity when transplanted into mice even at low clonal density and the ability to grow in culture in non-adherent conditions forming spheres are classically used to identify CSCs. Although it is still under debate whether tumor-initiating cell originates from the transformation of normal stem cells or the clonal evolution of genetically unstable cells with a capacity for the interconversion of different cellular states (Jordan, 2004; Sell, 2010), the concept of plasticity is central in CSC biology (Plaks et al., 2015). The plasticity of CSCs enables them to adapt and survive throughout biological stresses caused by the treatment and the continuous TME changes during tumor evolution, allowing dynamic and reversible transitions between quiescent and proliferative states, epithelial and mesenchymal states, differentiation, and metastasis (Plaks et al., 2015; Agliano et al., 2017; Müller et al., 2020; Romano et al., 2020). Under a persistently hostile environment that can develop at either the primary or metastatic tumor site, CSCs exploit evolutionarily conserved adaptation mechanisms and become dormant (Garimella et al., 2023), leading to a type of clinical remission, in which cancer cells are occult, undetectable, and asymptomatic for a variably protracted period, after which the tumor can recur in primary or distant sites (Enderling et al., 2013). The extreme variability and plasticity of CSCs due to genetic and epigenetic remodeling (Garimella et al., 2023) make their targeting challenging. ​

The maintenance of CSCs is ensured by adjacent cells in the TME, in particular by tumor-associated macrophages (TAMs) that form a specialized microenvironment that supports their survival against stress and injury and exerts a central role in maintaining their self-renewal and resistance characteristics. Tumor adaptation with the TME and interactions with TAMs throughout cancer progression can also lead differentiated tumor cells to take on CSC characteristics. (Ayob and Ramasamy, 2018).

Over the past decade, thanks to a deeper understanding of the CSC biology of resistant tumors, numerous efforts have focused on designing tailored therapies to target CSCs towards personalized medicine (Khan et al., 2015). However, the results obtained to date are far from conclusive and therapies against cancer stem cells remain an unmet goal (Cole et al., 2020).

In recent years, our understanding of cancer metabolic adaptations in a stressful TME has placed new hopes in modern cancer chemotherapy that can hinder CSCs with their dynamic cellular states by targeting the cornerstone of energy metabolism (Ayob and Ramasamy, 2018). Metabolic adaptations of CSCs and their supporting TAMs actually represent a demanding field of investigation. Our article deals with such an urgent field of investigation that may give new directions to cancer treatment. We review the latest studies that converge on the perception that mitochondrial function and OXPHOS metabolism meet the requirements of CSCs and their supporting TAMs from different tumor types. We offer an overview of therapies that disrupt the core of oxidative metabolism and, having shown a robust ability against CSCs in preclinical cancer studies, are currently studied in phase 1 and phase 2 clinical trials in their aspects of pharmacodynamics, pharmacokinetics, bioavailability, toxicity, together to efficacy on refractory and resistant tumors.

### CSCs and the mitochondrial respiratory machinery

The discovery that cancer has metabolic alterations dates back to the early 1920s when the biochemist Otto Warburg first proved that, oppositely to healthy cells, the metabolism of cancer cells mainly relies on glycolysis, uncoupled to OXPHOS, even under normal oxygen concentrations and fully functioning mitochondria. Tumor cells encompass hypoxia and re-oxygenation (Belisario et al., 2020), continuing their growth notwithstanding mutable environmental conditions. High lactate levels in the TME favor tumor acidosis and adaptation of cancer cells to hypoxia (Bononi et al., 2022). Hypoxia exerts a selection pressure that leads to the survival of subpopulations with the genetic machinery for malignant progression induced by HIF-1α and HIF-2α (Allavena et al., 2021). Lactate generated by glycolytic tumor cells is secreted outside the cell through the monocarboxylate transporter (MCT)4 and can be metabolized by adjacent cells (Martinez-Outschoorn et al., 2017). In oxygenated areas, lactate enters the tumor cell through MCT1 transporters and, upon conversion into pyruvate, produces the so-called “reverse Warburg phenotype” (Marchiq and Pouysségur, 2016). Pyruvate fuels the tricarboxylic acid (TCA) cycle and mitochondrial respiratory chain, increasing the NADH/NAD + ratio and mitochondrial biogenesis. In a physiological system of mouse adipocytes, Yang et al. showed that increased NADH/NAD + ratio induces Sirtuin 1 (SIRT1)-mediated deacetylation of the peroxisome proliferator-activated receptor gamma coactivator-1α (PGC-1α), leading to activation of such a pivotal promoter of mitochondrial biogenesis (Yang et al., 2020).

Several studies highlight expression of MCT trasporters in different cancer settings. Using varied tumor mouse models (colorectal adenocarcinoma, human cervix squamous cell carcinoma, hepatocarcinoma, lung adenocarcinoma), Sonveaux et al. found MCT1 expressed on a subset of resistant cancer stem-like cells and its targeting had clinical antitumor potential (Sonveaux et al., 2008). They demonstrated that MCT1 inhibition induced a switch from lactate-fueled respiration to glycolysis, which overcame cancer resistance and induced sensitivity to ionizing radiation (Sonveaux et al., 2008). Curry et al. interrogated head and neck cancer (HNSCC) tissues to assess metabolic compartmentation in primary tumors typically composed in upper layer of differentiating squamous carcinoma cells and a basal stem cell layer that regenerates the tumor. The basal layer was mitochondrial-rich and specialized for the use of mitochondrial fuels, such as L-lactate and ketone bodies and expressed high levels of MCT1. Conversely, the majority of well-differentiated carcinoma cells and cancer-associated fibroblasts (CAFs) showed strong MCT4 immunoreactivity (Curry et al., 2013).

Pancreatic ductal adenocarcinoma (PDAC) cells do express MCT1 and MCT4 (Kong et al., 2016). Through immunohistochemistry of PDAC tissues, Sandforth et al. demonstrated a co-localization of MCT1 with KLF4 (Sandforth et al., 2020). Moreover, they demonstrated that MCT1 expression on PDAC cell lines conferred greater potential of clonal growth, along with drug resistance and elevated expression of the stemness marker nestin and reprogramming factors (OCT4, KLF4, NANOG). These effects on stemness properties were abrogated by targeting of MCT1 (Sandforth et al., 2020). Pancreatic CSCs, defined using spheres and enriched through CD133 marker, were also shown to express increased levels of PGC-1α, demonstrated to be a relevant determinant of their OXPHOS dependency (Sancho et al., 2015). PGC-1α forced expression in CD133 pancreatic cancer cells accelerated OXPHOS metabolism and enabled their self-renewal and tumorigenic capacity (Valle et al., 2020).

MCT1 and MCT4 are expressed in glioblastoma tumors (Park et al., 2018). Takada et al. measured an upregulation of MCT1 along with stem cell markers (Nestin, NANOG, CD133, SOX-2, and OCT-4) in sphere-forming glioblastoma cells compared with adherent, non-sphere forming cells. Inhibition of MCT1 decreased the viability of glioblastoma CSCs compared with that of non-CSCs (Takada et al., 2016). Mudassar et al. showed MCT1 transporters were associated with high mitochondrial abundance in high grade glioma cells (Mudassar et al., 2020). PGC-1α suppression hampered spheroid formation of glioblastoma cells *in vitro* and their capability to form *in vivo* tumors (Bruns et al., 2019). Other studies, associate PGC-1α with cancer metastasis and resistance (Vazquez et al., 2013; LeBleu et al., 2014). PGC-1α expression was co-induced with EMT genetic program in breast cancer patients with distant metastasis and poor outcome (LeBleu et al., 2014). Also, PGC-1α supports high bioenergetic and ROS detoxification capacities of resistant melanoma tumors with higher rates of survival under oxidative stress compared to PGC-1α-negative melanomas (Vazquez et al., 2013). Mitochondrial biogenesis is essential for the anchorage-independent survival and propagation of stem-like cancer cells (De Luca et al., 2015). For a review of MCT transporters in cancer and the potential of new selective MCT1 and/or MCT4 inhibitors in cancer therapeutics, we refer to Singh et al. (Singh et al., 2023).

Evidence that oxidative phosphorylation is upregulated in CSCs is increasingly emerging (Abdullah and Chow, 2013; Sancho et al., 2016; Li et al., 2020; Karp and Lyakhovich, 2022). Studying one of the most aggressive and resistant cancers, i.e., pancreatic ductal adenocarcinoma, Viale et al. found that a subpopulation of dormant tumor cells responsible for tumor relapse relied on oxidative phosphorylation for survival and had features of cancer stem cells (CD133^+^CD44^high^ cells with spherogenic and tumorigenic capabilities) (Viale et al., 2014). Valle et al. by changing the carbon source from glucose to galactose *in vitro*, induced a forced oxidative metabolism in pancreatic cancer cells (Valle et al., 2020). Such a metabolic switch produced enrichment in typical pancreatic CSC biomarkers (Hermann et al., 2007) including pluripotency gene expression, tumorigenic potential, upregulated immune evasion properties and acquisition of plastic features such as a reversible quiescence-like state (Valle et al., 2020). Dependency on mitochondrial metabolism has been demonstrated in CSCs from ovarian cancer, identified through coexpression of CD44 and CD117 and tumor-initiating capacity (Pastò et al., 2014). In ovarian cancer patients, comparative transcriptome analyses from ascites-derived tumor cell spheroids *versus* tumor samples revealed upregulation of genes involved in oxidative phosphorylation process along with those of chemoresistance, cell adhesion and cell-barrier integrity (Ding et al., 2021).

In small cell lung cancer, resistant CSC-like cells, identified based on selective expression of urokinase-type plasminogen activator receptor (uPAR^+^), showed higher dependency on oxidative phosphorylation than non-CSCs (uPAR^−^) (Gao et al., 2016). The glycosylphosphatidylinositol (GPI)-anchored protein uPAR is associated with multidrug resistance and with high clonogenic activity (Gao et al., 2016). Vlashi et al. showed that stem/progenitor cells from neurospheres depended on oxidative phosphorylation and higher ATP content compared with differentiated glioblastoma cells derived from culture in monolayers (Vlashi et al., 2011). They also show that such a OXPHOS dependence is lost during differentiation and accompanied with a switch to aerobic glycolysis (Vlashi et al., 2011). Evidence that mitochondrium is a relevant target to overcome resistance of colorectal CSCs are reviewed by Rainho et al. (Rainho et al., 2023). Following metabolic profiling of primary chronic myeloid leukemia (CML) cells, Kuntz et al. found a three-fold increase in the rate of mitochondrial oxygen consumption along with a pattern of metabolites indicating increased lipolysis and fatty acid oxidation in the stem cell-enriched population (CD34^+^CD38^−^), compared to differentiated CML cells (CD34^−^) (Kuntz et al., 2017). Inhibition of oxidative phosphorylation by tigecycline, an anti-bacterial FDA-approved antibiotic, produced a selective cytotoxic effect on CSC at clinically administrable doses (Kuntz et al., 2017). This study highlights that although the nature of CSCs differs and different origins of CSCs are postulated between hematological and solid tumors (Bonnet and Dick, 1997; Jordan, 2004), the requirements of CSCs appear to be met by oxidative metabolism across different tumors.

**FIGURE 1:**
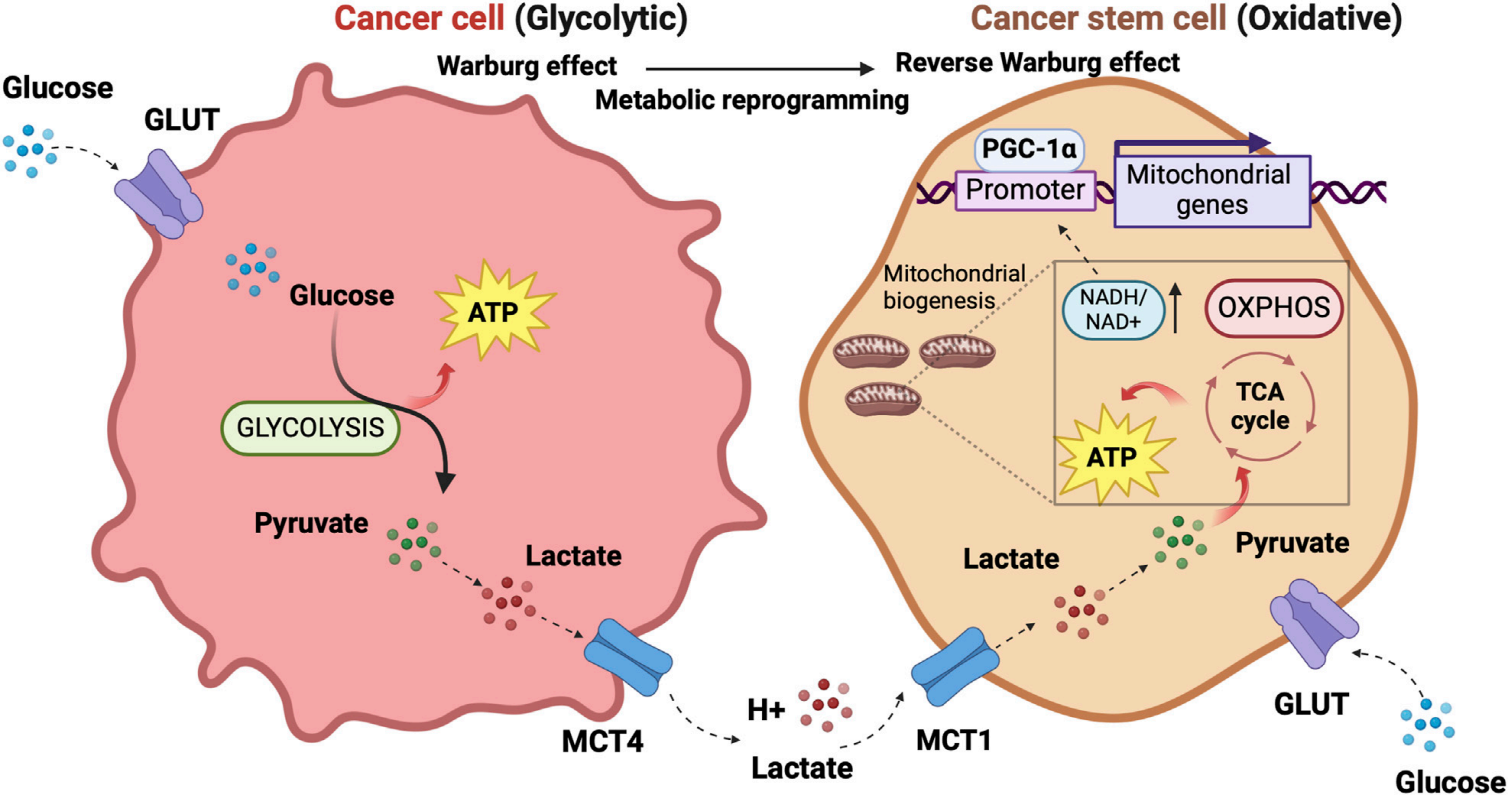
The prevalent metabolism adaptations in differentiated tumor cells compared to stem cell-like tumor cells support a metabolic symbiosis between the different cellular states. The illustration was started from scratch, created with BioRender.com original design.

The concept of metabolic symbiosis between hypoxic/glycolytic- and OXPHOS-tumor cells that favors rapid adaptation of cancer to changing environmental oxygen conditions (Nakajima and Van Houten, 2013) can virtually unravel an interplay between non-CSCs and CSCs in which differentiated tumor cells provide glycolysis products that fuel oxidative metabolism of the stem, regenerative and resistant cellular component of the tumor. (Figure 1).

### OXPHOS and multidrug resistance

Increased activity of ATP binding cassette (ABC) transporter family members involved in multidrug resistance is a common feature of CSCs (Begicevic and Falasca, 2017). There are several efforts focused on creating druggable molecules to inhibit these transporters. Five-cyano-6-phenylpyrimidin derivatives containing an acylurea moiety demonstrated efficacy in inhibiting P-glycoprotein ABCB1, a leading member of ABC transport proteins found to be widely overexpressed in human solid tumors and hematologic malignancies (Wang et al., 2018a; Wang et al., 2018b). ABC transporters are highly dependent on ATP since they use the energy from ATP hydrolysis to pump substrates out of cells (Linton and Higgings, 2007). ATP generated by the respiration of mitochondria in the proximity of the plasma membrane and transported from the mitochondrial matrix to the cytosol nearby plasma membrane produces a local rise of ATP level for the active transporter’s need (Linton and Higgings, 2007), thus explaining why mitochondrial and not glycolytic ATP preferentially fuels ABC transporter activity in chemoresistant cancer cells (Linton and Higgings, 2007). In a model of chemoresistant cancer cells, Giddings et al. found that methylation-controlled J protein (MCJ) affected ABC transporter function through regulation of mitochondrial respiration (Giddings et al., 2021). MCJ localizes on the inner membrane of mitochondria and negatively regulates Complex I thus acting as an endogenous brake on mitochondrial respiration (Hatle et al., 2013) As MCJ is often downregulated in the tumors, the authors generated MCJ mimetics and investigated their capability to inhibit ABC transporter function and therapeutic efficacy in combination with doxorubicin, using ovarian and mammary cancer cells and an *in vivo* mouse model of mammary tumor (Giddings et al., 2021). MCJ mimetics attenuated mitochondrial respiration in chemoresistant cells and reversed cancer chemoresistance *in vivo* tumor model MCJ-KO. The tumors of mice treated with a combination of MCJ mimetics and doxorubicin showed a prominent size reduction compared to those treated with doxorubicin alone. There was no evidence of liver and heart toxicity by MCJ mimetics nor effect on mouse body weight (Giddings et al., 2021). Although not selectively involving CSCs, the study by Giddings et al. sheds light on the aspect of chemoresistance closely linked to the stem-cell-like concept.

### OXPHOS and tumor dormancy

Dormancy is a strategy adopted by a tumor cell placed in a persistently hostile environment that exploits evolutionarily conserved adaptation mechanisms to succeed in tumor progression (Merlo et al., 2006). Proliferation arrest, metabolic quiescence, and immune occultation are the main features of tumor dormancy (Enderling et al., 2013). Dormant cancer cells can reawaken in response to signals which are not yet fully understood, resulting in recurrence and metastasis (Gao et al., 2016; Park and Nam, 2020). Adapting newly arrived cancer cells to the microenvironment of distal organs is a stringent rate-limiting step in metastasis, and the probability of completing this step varies widely depending on the tumor type and the target organ. A study of the metabolic signature associated with disseminated cancer cells suggested an activation of mitochondrial bioenergetic pathways (TCA cycle and OXPHOS) and the pentose-phosphate pathway (Dudgeon et al., 2020) upon seeding. Newly seeded cancer cells slow down bioenergetics and become dormant to survive in secondary sites (Ganguly and Kimmelman, 2023). Although how and when dormant tumor cells become reactivated after inactivity remains not well understood, a role for lipid metabolism in reawakening is emerging (Luo et al., 2017; Watt et al., 2019). Pascual et al. (Pascual et al., 2017) found a subpopulation of CD44bright slow-cycling cells in human oral carcinomas with a unique ability to initiate metastasis that expressed high levels of the fatty acid receptor CD36 and lipid metabolism genes (Pascual et al., 2017). Using neutralizing antibodies for CD36 blockade, they were able to inhibit metastasis formation in orthotopic mouse models of human oral cancer. Conversely, palmitic acid or a high-fat diet increased the metastatic potential of CD36^+^ cancer cells (Pascual et al., 2017). Ladanyi et al. demonstrated a role for adipocytes in the stimulation of CD36 and Fatty acid transport protein 1 (FATP1) in ovarian cancer cells (Ladanyi et al., 2018) suggesting a significant role for cancer-associated adipocytes in tumor growth and metastasis through favoring lipid utilization and uptake and metabolic reprogramming (Yao and He, 2021). Intriguingly, the oxidation of Cys272 and Cys333 promoted the activation of CD36, suggesting a regulatory effect of the redox signaling in the reactivation of dormant cancer cells (Wang et al., 2019). Also, oxidative stress enabled P450 epoxygenases to synthesize epoxyeicosatrienoic acids, metabolites of arachidonic acid, with a vasodilation effect facilitating exit from the dormant state (Borin et al., 2017).

### The CSC niche and tumor associated macrophages

Adjacent cells to CSC form a specialized microenvironment, termed niche, essential for preserving and sustaining CSC against stress and injuries with growth factors, cytokines, and extracellular matrix compounds (Allavena et al., 2021). In analogy to the physiological stem cell niche, this specialized tissue structure allows CSCs to survive and remain quiescent and also provides cues for reactivation of proliferation, differentiation, and migration. (Allavena et al., 2021).

CSCs niche dynamics vary between leukemia and solid tumors. Tracing the cellular origins of human cancers has long been a complex and contentious area in cancer research. Pioneering work by John Dick and colleagues in the 1990s introduced the hierarchical model in acute myeloid leukemia (AML), proposing that a primitive stem or early progenitor cell serves as the cell of origin for malignant transformation in AML (Lapidot et al., 1994; Bonnet and Dick, 1997). This model delineates a rare population of leukemic stem cells (LSCs) with high self-renewal potential and immunophenotypic resemblance to healthy hematopoietic stem cells (HSCs), which are exclusively capable of reinitiating leukemia in immunodeficient mice (Lapidot et al., 1994; Bonnet and Dick, 1997). In leukemia, the bone marrow serves as primary niche, populated by healthy stem cells with which CSCs compete (Marchand and Pinho, 2021). The leukemic niche is populated by different cell types, such as mesenchymal stem cells (MSCs), endothelial cells, megakaryocytes, macrophages, osteoblasts, and nerve cells (Schepers et al., 2015). Bidirectional interactions between leukemic cells and the bone marrow microenvironment promote leukemic progression at the expense of healthy hematopoiesis, implicating bone marrow mesenchymal stem cells in the predisposition, manifestation, and evolution of hematological malignancies (Korn and Méndez-Ferrer, 2017).

In contrast, solid tumors exhibit phenotypic plasticity, where tumor cells can can interconvert between differentiated and stem-like states across a continuum of cell fate specification (Quail et al., 2012). Moreover, despite the presence of founder mutations within the parental clones, a large number of additional mutations between primitive and metastatic tumor implicate the concept of clonal evolution in CSC development (Campbell et al., 2010; Kreso and Dick, 2014). The fact that melanoma, breast, prostate, ovarian, and lung cancer cells are all able to alter their gene expression to resemble cell types that are not part of their original lineage (Quail et al., 2012) exemplifies cancer cell plasticity that enables cancer cells to gain/lose stem cell properties (Passalidou et al., 2002; Shirakawa et al., 2002; Lim et al., 2009). Solid tumors contain non-tumor stromal cells supporting CSCs including CAFs, MSCs, TAMs and other immune cells, and extracellular matrix proteins (Mancini et al., 2021). The niche is characterized by conditions of hypoxia, acidity, and low glucose levels (Olivares-Urbano et al., 2020). The niche concept extends to specialized pre-metastatic microenvironments that play a crucial role in the colonization of disseminated tumor cells at secondary sites, with organ-specific exosomes derived from primary tumors facilitating colonization (Fong et al., 2015; Hoshino al.al 2015). Once the pre-metastatic niche has finished priming, the metastatic niche generates a microenvironment that sustains metastatic cancer stem cells, providing physical anchorage, survival, immune surveillance protection, and metabolic requirements for CSCs in distant metastatic sites (Joseph et al., 2023).

TAMs are the leading players in the CSC niche, they physically interact with CSCs and secrete a variety of soluble factors to protect them from environmental damage (Jinushi et al., 2011; Fan et al., 2014; Zhou et al., 2015; Oshimori, 2020). Notably, similarities exist between TAMs from leukemias and solid tumors within their respective niches. Such similarities consist in abundant localization of TAMs in both leukemic and solid tumor niches that positively correlate with CSC distribution (Wang and Zheng, 2019; Basak et al., 2023) and accumulation within hypoxic tumor regions, where CSCs are also prevalent (Wang and Zheng, 2019; Basak et al., 2023). CSCs exert significant influence over TME by recruiting and polarizing macrophages toward a pro-tumor M2 phenotype. In turn, M2-TAMs actively support CSC maintenance, thus promoting a symbiotic relationship between these cellular populations (Wang and Zheng, 2019; Basak et al., 2023).

TAMs are able to activate signaling pathways essential to CSCs, including those driven by Sonic Hedgehog (SHH), Neurogenic locus notch homolog protein (NOTCH), STAT3, PI3k/Akt, Wingless integrated (WNT)/b-catenin and NANOG, through soluble factors or direct physical interaction with CSC (Allavena et al., 2021). Tumor cells produce chemotactic factors (Allavena et al., 2021), exosomes (Su et al., 2021) and metabolites (Diskin et al., 2021) to recruit circulating monocytes and tissue-resident macrophages and induce their polarization towards anti-inflammatory, angiogenic and protumor (M2) phenotype typical of TAMs. TAMs initiate reciprocal crosstalk with CSC to exert their trophic action in the niche (Allavena et al., 2021). Transcription factors involved in maintaining the pluripotency and self-renewal characteristics of CSCs are highly expressed by TAMs (Shang et al., 2023). The CSC’s role in modulating the TME and driving the recruitment and alternative polarization of macrophages and crosstalk between CSCs and TAMs have been extensively reviewed in several articles (Sainz et al., 2016; Muller et al., 2020; Allavena et al., 2021; Chae et al., 2023).

**FIGURE 2:**
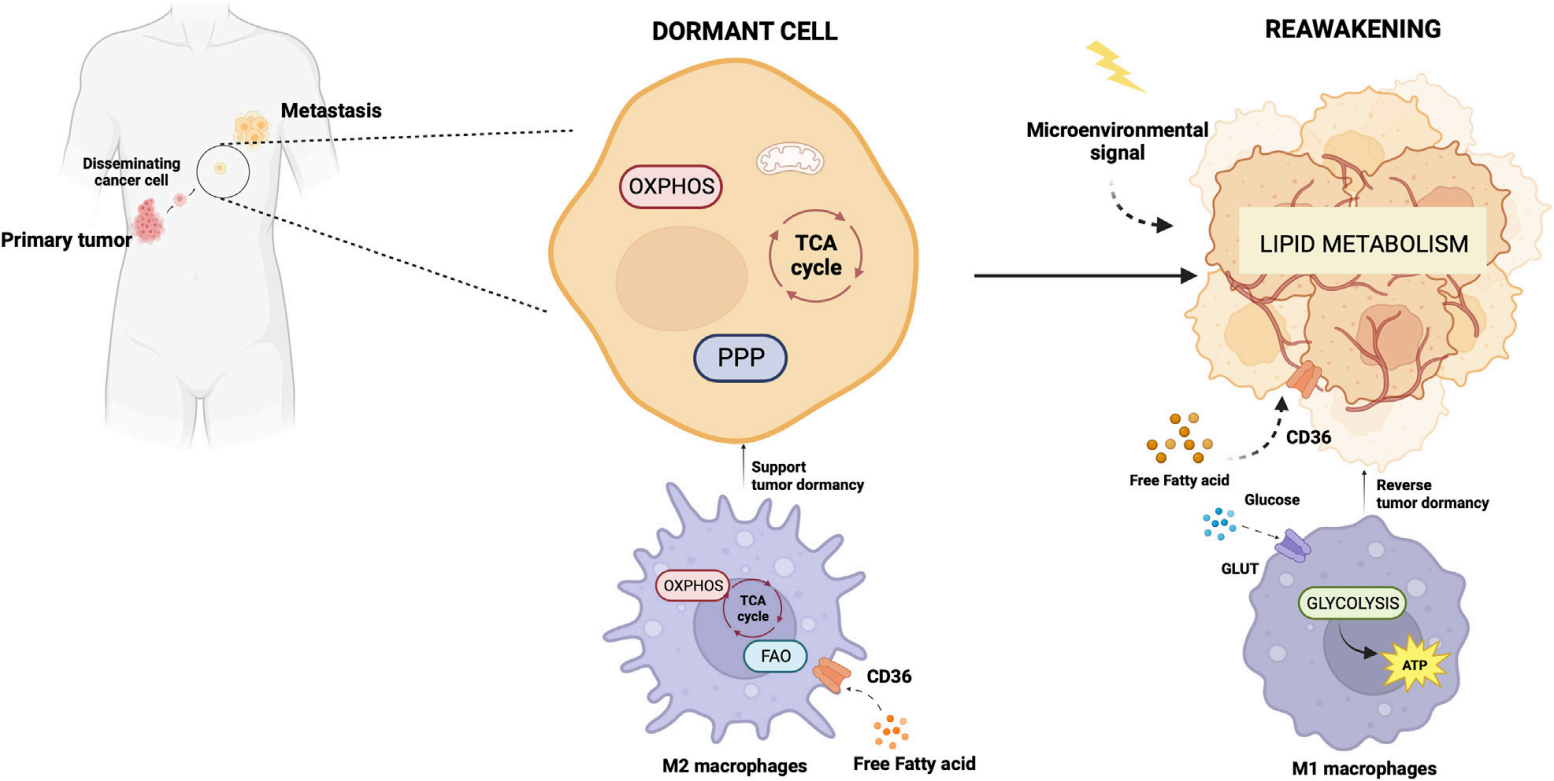
Metabolic aspects of TAMs in tumor dormancy and reawakening. The illustration was started from scratch, created with BioRender.com original design.

The primary tumor secretome influences the immune milieu at distant organs, thus preparing the permissive soil for colonization of disseminated cancer cells by re-educating the metabolic and epigenetic state of resident cells in the host organs (Ganguly and Kimmelman, 2023). Macrophages play a special role in priming and disseminating tumor cells for dormancy and stemness (Borriello et al., 2022). Using a technique termed Window for High-Resolution Imaging of the Lung (WHRIL) (Entenberg et al., 2018), Borriello et al. quantitatively measured, in real-time, spontaneously disseminating tumor cells during the process of metastasis to the lung, in a breast cancer mouse model (Borriello et al., 2022). They found a subset of macrophages within the primary tumor that caused activation of genetic programs related to dissemination, dormancy, and stemness in tumor cells approaching the intravasation site. Upon tumor cell contact with macrophages, tumor cell expresses high levels of the actin-regulatory protein MenaINV (Roussos et al., 2011). This actin isoform plays an active role in tumor cell migration during intravasation within the primary tumor (Pignatelli et al., 2016). Moreover, contact with macrophages activated in tumor cells expression of stem-like SOX-9 phenotype and Nuclear Receptor Subfamily 2 Group F Member 1 (NR2F1), the orphan nuclear receptor and one of the best molecular markers of dormancy that regulates expression of pluripotency genes (Sosa et al., 2015). The depletion of macrophages significantly reduced NR2F1 levels in the tumor cells and prevented dormancy (Borriello et al., 2022). Before disseminating, tumor cells establish microenvironmental niches incorporating macrophages in the primary tumor that enable them to acquire a pro-dissemination, stem-like dormancy phenotype that is carried to the secondary site and is lost during metastatic growth (Borriello et al., 2022). Dormancy represents a typical risk for long-term breast cancer survivors. Dormant breast cancer cells preferentially reside in the bone marrow. A study by Walker et al. in a breast cancer mouse model showed that bone marrow M2 macrophages supported tumor dormancy. Upon the M2 to M1 switch through activation of TLR4 with LPS, M1 macrophages reversed dormancy and induced sensitivity to carboplatin of breast cancer cells (Walker et al., 2019). The authors demonstrated that M1-derived exosomes produced clinical evidence of metastasis due to the activation of NF-κB in quiescent breast cancer cells to reverse non-cycling to cycling cells (Walker et al., 2019). Crosstalk between macrophages and dormant cancer cells has been extensively reviewed by Batoon and McCauley (Batoon et al., 2021). Figure 2 illustrates the interplay between dormant cell and macrophages.

### The mitochondrial respiratory machinery is a significant driving force in TAM polarization

Phenotype, function, and metabolic state are closely interconnected aspects in macrophages and coordinated with each other (Minhas et al., 2019; Emtenani et al., 2022; Gonzalez et al., 2023). Through single-cell transcriptomic profiling of macrophages phagocytosing neoplastic cells, Gonzales et al. demonstrated a strict linkage between phagocytosis, immune-suppressive phenotype, and gene expression changes toward OXPHOS, ribosomal, and other metabolic genes (Gonzalez et al., 2023). The correlation of the metabolic gene signature with worse clinical outcomes was validated in human lung cancer (Gonzalez et al., 2023). Consistent with the findings by Gonzales et al., Minhas et al. showed that genetic or pharmacological blockade of *de novo* NAD + synthesis, suppressed mitochondrial NAD + -dependent signaling and respiration, and impaired phagocytosis and resolution of inflammation due to changes in macrophage polarization state. (Minhas et al., 2019). Emtenani et al. (Emtenani et al., 2022), investigating gene expression in macrophages during the first migratory stages of the tissue invasion, found a metabolic reprogramming towards OXPHOS and ribosome biogenesis of migrating macrophages. In this cell model, the authors identified Atossa, a transcriptional regulator inducing expression of an RNA helicase termed Porthos. This factor increased the translation efficiency of short 5′UTR mRNAs that included a subset of mitochondrial OXPHOS genes of the respiratory complexes (Emtenani et al., 2022).

**FIGURE 3:**
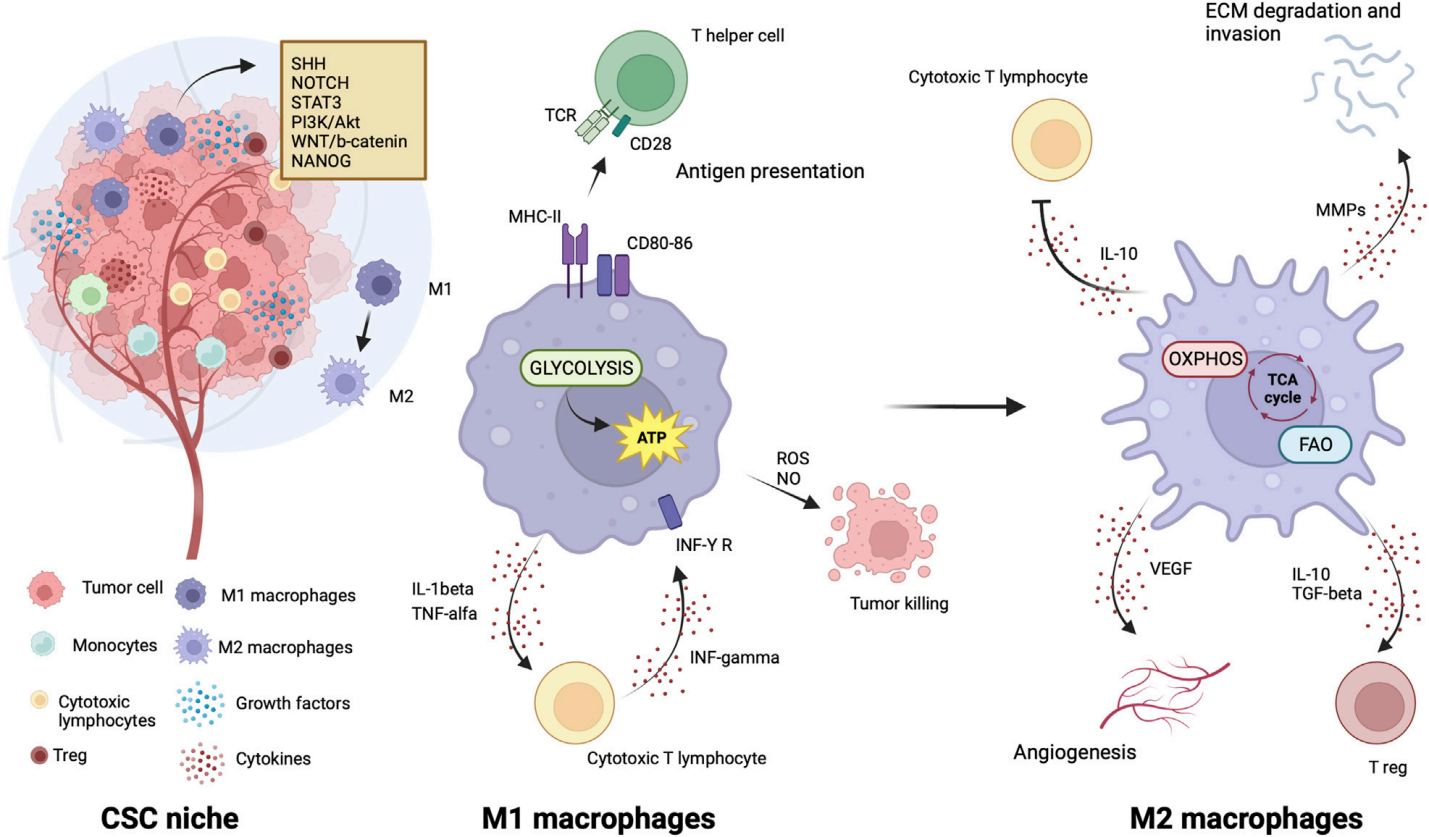
Metabolic features of TAMs in CSC niche. The illustration was started from scratch, created with BioRender.com original design.

Like CSCs, M2 macrophages can resist and remain functional in adverse environmental conditions such as low nutrients, low pH, hypoxia, and oncometabolite abundance (Liu et al., 2021). Like CSCs, M2 macrophage metabolism exploits the mitochondrial respiratory machinery that is a significant driving force in alternative macrophage polarization (O'Neill et al., 2016; Liu et al., 2021). While aerobic glycolysis produces most of the ATP and intermediates for biosynthetic pathways required for effector (microbicidal and antitumor) functions of M1 macrophages (Warburg and Minami, 1923; Altenberg and Greulich, 2004; Tannahill et al., 2013), to sustain their activities, M2 macrophages use the TCA cycle to obtain reducing equivalents, assuring constant energy production in concert with mitochondrial OXPHOS (Figure 3). Acetyl-CoA oxidized in the TCA cycle mainly derives from fatty acid oxidation (Odegaard and Chawla, 2011; O'Neill et al., 2016). M2 macrophages actively extract fatty acids from circulating lipoproteins internalized through CD36 (Evans et al., 1993) and endocytosis (Huang et al., 2014). The pivotal role of fatty acids oxidation in alternative macrophage polarization is underscored by the observation that blocking palmitate entry into the mitochondrial matrix hampers IL-4-induced M2 polarization (Malandrino et al., 2015). Enhanced fatty acid oxidation in palmitate-incubated macrophages reduced the inflammatory profile (Malandrino et al., 2015). Proliferator-activated receptors of peroxisomes, organules involved in the oxidation of long-chain fatty acids and eicosanoid-CoA esters (Reddy and Hashimoto, 2001) were shown to regulate the transcription of M2 genes, (Odegaard et al., 2008; Chawla, 2010; Nelson et al., 2018).

### OXPHOS-targeted drugs in clinical trials

Efforts in recent years led to design of numerous clinical trials for the assessment of the anticancer effectiveness of drugs targeting mitochondrial metabolism with the aim of hindering CSCs and microenvironmental signaling together.

The electron transport chain has indepth been explored for development of inhibitors for each complex (Sainero-Alcolado et al., 2022). However, these compounds can exhibit remarkable toxicity that prevents their use in clinical practice (Sainero-Alcolado et al., 2022). The antidiabetic agent metformin (Watanabe, 1918) counts more than 400 registered clinical trials in the last 5 years as a cancer chemopreventive or therapeutic agent, alone or in combination with neoadjuvant chemo-radiation therapy (https://www.clinicaltrials.gov). The master pathway of metformin anticancer activity is the activation of the adenosine monophosphate-activated protein kinase (AMPK) that inhibits mammalian target of rapamycin (mTOR) (Zhou et al., 2001) pathway triggered by inhibition of complex I through drug binding in the quinone channel (Owen et al., 2000). Recently, metformin was found to cause a mitochondrial effect independent of inhibition of complex1 by direct molecular targeting PEN2, a subunit of γ-secretase (Ma et al., 2022). PEN2 binds to ATPase H^+^transporting accessory protein 1, inhibits the activity of ATPase without increasing AMP or ADP, and then activates the lysosomal AMP-independent AMPK pathway (Ma et al., 2022). Still, other mechanisms concur to its anticancer activity that are still not well understood. Metformin reduces cancer risk, decreases cancer-related mortality in patients with diabetes (Decensi et al., 2010), and has excellent performance in preclinical studies. Particularly, a preclinical study shows that metformin selectively targets cancer stem cells and acts together with chemotherapy to block tumor growth and prolong remission (Hirsch et al., 2009). A prospective phase I clinical trial (NCT01442870) assessing the safety of metformin in combination with chemotherapy in patients with solid tumors suggests that metformin can be given safely with chemotherapy (Saif et al., 2019). Brown et al. evaluated the impact of metformin on CSC number and clinical outcomes in nondiabetic patients with advanced-stage epithelial ovarian cancer. Metformin decreased by 2.4-folds the number of ALDH+CD133+ CSCs and increased sensitivity to cisplatin *ex vivo*. Translational studies confirm an impact of metformin on ovarian cancer CSCs and suggest epigenetic change in the tumor stroma, specifically MSCs, may drive the platinum sensitivity *ex vivo*. Metformin treatment was associated with increased overall survival, supporting the use of metformin in phase III studies (Brown et al., 2020). However, benefits in cancer treatment are often quite vague in clinical trials; thus, there are challenges in the clinical translation of metformin. In a very recent review, Hua et al. (Hua et al., 2023) point out that the mechanisms of action of metformin must be seen in the context of cancer hallmarks, the well-standardized set of crucial functional capabilities for malignant transformation (Hanahan, 2022). In their article, after summarizing the current knowledge on the antitumor action of metformin, the authors elaborate the underlying mechanisms in terms of cancer hallmarks and propose new perspectives of metformin use potentially applicable to cancer treatment (Hanahan, 2022).

IACS-010759, an inhibitor of complex I, was found to reduce mitochondrial function of enriched tumor cell spheroids from the ascites of high-grade serous ovarian cancer patients. Also, IACS-010759 treatment reduced the fraction of CD34^+^ progenitor AML cells in a dose-dependent manner (Molina et al., 2018). Current clinical trials with IACS-010759 involve advanced tumors (phase 1, NCT03291938) and AML (phase 1, NCT02882321). Tamoxifen was found to interact with the flavin mononucleotide site of complex I leading to mitochondrial failure (Moreira et al., 2006). It is investigated in cancers other than breast, and genito-urinary tract, as intraocular melanoma, in combination with cisplatin (phase 2, NCT00489944); high risk stage III melanoma in combination with sorafenib (phase 2, NCT00492505); oesophageal cancer (phase 1, NCT02513849); osteosarcoma (phase 1, NCT00001436). Pyrvinium pamoate is a lipophilic cation belonging to the cyanine dye family, inhibiting complex I. (Schultz and Nevler, 2022). It has been used in the clinic as a safe and effective anthelminthic for over 70 years (Schultz and Nevler, 2022) and currently is investigated in pancreatic cancer to determine its safety and tolerability (phase 1, NCT05055323). Atovaquone, with a structure similar to protozoan ubiquinone, is an inhibitor of complex III (Mather et al., 2005) approved by the US Food and Drug Administration against plasmodium falciparum. Atovaquone reduced the tumorsphere formation and invasion ability of EpCAM^+^CD44^+^ CSCs isolated from HCT-116 colon carcinoma cell lines (Fu et al., 2020). It was found to inhibit proliferation and induce apoptosis of CSCs (CD44^+^CD24Low^−^ and ALDH^+^) derived from the mammary breast cancer cell line MCF7 (Fiorillo et al., 2016b) and of ALDH^+^CD133^+^ cancer stem-like cells from two high-grade serous ovarian cancer patients (Kapur et al., 2022). Atovaquone anti-cancer efficacy has been assessed in varied mouse cancer models (Rodriguez-Berriguette et al., 2024) and is currently investigated in NSCLC (phase 1, NCT04648033), ovarian cancer (phase 2, NCT05998135), AML (phase 1, NCT03568994). Niclosamide is an uncoupler of electron transport chain (Chen et al., 2018). Jin et al., showed that niclosamide is a potent inhibitor of the NF-κB pathway and exerts a synergism with Ara-C or VP-16 against primary AML cells. They also suggested that this drug has the potential to eradicate AML blasts since they demonstrated that niclosamide kills AML CD34^+^CD38^−^stem-cells, while sparing normal bone marrow progenitors (Jin et al., 2010). Niclosamide efficiently decreased therapy resistance in colorectal cancers by reducing CSC populations and their self-renewal activity, thereby attenuating the survival potential of CSCs following chemoradiation (Park et al., 2019). Clinical trials with niclosamide involved treatment of refractory AML (phase 1, NCT05188170), colorectal cancer (phase 1, NCT02687009; phase 2, NCT02519582), and castration resistant prostate cancer (phase 1, NCT03123978; phase1, NCT02532114; phase 2, NCT02807805). Table 1 lists active or recently completed clinical trials investigating outcomes with respiratory-complex inhibitors in refractory tumors, ONC201 and ONC206 are imidazo-pyrido-pyrimidine derivatives that bind to the mitochondrial serine protease termed caseinolytic protease proteolytic subunit (ClpP) with the ability to reduce mitochondrial oxidative phosphorylation, oxygen consumption rate, ATP production and increase mitochondrial generation of reactive oxygen species (Przystal et al., 2022). They were found for the first time to affect mitochondrial activity in diffuse midline glioma cells in children and young adults and considered two promising agents against Histone three lysine27-to-methionine (H3.3K27M)-mutated gliomas. Treatment with ONC201 reduced self-renewal, clonogenicity and cell viability of GBM cells (He et al., 2021). Similar results of inhibition of tumorsphere formation, CSC genes NANOG and SOX2, and CSC frequency were obtained by Jeon et al., due to selective antagonism of dopamine receptor (Jeon et al., 2023). Moreover, ONC201 targets chemotherapy-resistant colorectal cancer stem-like cells (Prabhu et al., 2015) and significantly decreased CSC frequency and tumor initiation capability in a breast cancer mouse model (Greer et al., 2022). In chemo-refractory AML patient samples, ONC201 induced apoptosis in leukemia stem/progenitor cells (CD34^+^/CD38^-^) to an extent that was equivalently observed in non-CSCs (Ishizawa et al., 2016) Especially ONC201 is an investigational agent that has shown a favorable safety profile in phase 1 and phase 2 clinical trials in advanced cancers. Several clinical trials have been designed to assess efficacy of ONC201 and ONC206, alone or in combination with chemo or immunotherapy, against several cancer types, including colorectal cancer, pediatric H3. K27M-mutant gliomas, adults with recurrent H3.K27M-mutant gliomas, recurrent gliomas, rare primary central nervous system neoplasms, neuroendocrine tumors, multiple myeloma, endometrial cancer, advanced solid tumors, metastatic breast cancer, relapsed/refractory non-Hodgkin’s lymphoma, relapsed or refractory acute leukemias, oral cancer (Table 2). Two completed clinical trials (NCT02250781, NCT02324621) evaluated the safety, pharmacokinetics, and pharmacodynamics of ONC201 in patients with advanced solid tumor that is refractory to standard treatment, or for which no standard therapy is available. Results from these studies indicated that oral ONC201 is well-tolerated and had immunostimulatory activity. Patients treated with ONC201, who experienced at least stable disease by RECIST for 12 or more weeks, broad induction of immune cytokines and effector molecules was observed (Stein et al., 2019). Also, increased intratumoral infiltration of cytotoxic NK cells and granzyme B was observed in a metastatic prostate cancer patient in response to ONC201.

**TABLE 1:** Clinical trials investigating the outcomes of the treatments of refractory tumors with inhibitors of mitochondrial respiratory complexes.

Title	ClinicalTrials. gov ID	Phase	Primary outcome measure	Study completion, actual/estimated	Enrollment, actual/estimated
Oxidative Phosphorylation Inhibitor IACS-010759 in Treating Patients With Relapsed or Refractory Acute Myeloid Leukemia	NCT02882321	1	Maximum tolerated dose	2022–04	17
			Clinical response (duration, progression free survival, overall survival)	The study terminated for apparent lack of effectiveness	
A Study to Determine if the Drug, Pyrvinium Pamoate, is Safe and Tolerable in Patients With Pancreatic Cancer	NCT05055323	1	Safety and tolerability	2024–04	18
			Pharmacokinetic, pharmacodynamic profile and bioavailability in humans		
Atovaquone With Radical ChemorADIotherapy in Locally Advanced NSCLC (ARCADIAN)	NCT04648033	1	Dose limiting toxicity; maximum tolerated dose; recommended phase II dose	2023–10	21
Repurposing Atovaquone for the Treatment of Platinum-Resistant Ovarian Cancer	NCT05998135	2	Progression free survival	2025–06	28
Atovaquone (Mepron^®^) Combined With Conventional Chemotherapy for *de Novo* Acute Myeloid Leukemia (AML) (ATACC AML)	NCT03568994	1	Atovaquone plasma levels at time points including bone marrow assessment. Toxicity and steady state concentrations when given in combination with standard chemotherapy	2025–10	26
Niclosamide in Pediatric Patients With Relapsed and Refractory AML	NCT05188170	1	Dose-limiting toxicity; clinical response	2026–12	16
Enzalutamide and Niclosamide in Treating Patients With Recurrent or Metastatic Castration-Resistant Prostate Cancer	NCT03123978	1	Safety and recommended dose	2022–04	6
Abiraterone Acetate, Niclosamide, and Prednisone in Treating Patients With Hormone-Resistant Prostate Cancer	NCT02807805	2	PSA response rate; dose limiting toxicity; clinical response	2024–06	37

**TABLE 2:** Clinical trials investigating the outcomes of tumor treatments with ONC2091 and ONC206 (Imipridones).

Title	ClinicalTrials. gov ID	Phase	Primary outcome measure	Study completion, actual/estimated	Enrollment, actual/estimated
Testing ONC201 to Prevent Colorectal Cancer	NCT05630794	1	To determine the optimal cancer preventive dose of ONC201	2028-01-01	24
ONC201 in Pediatric H3 K27M Gliomas	NCT03416530	1	Determination of recommended Phase 2 dose, as a single agent or in combination with radiation	2023-09-30 active	130
ONC201 and Atezolizumab in Obesity-Driven Endometrial Cancer	NCT05542407	1	Determination of recommended phase 2 dose in combination with Atezolizumab; tumor response according to RECIST Criteria	2025-01-15	58
ONC201 in Adults with Recurrent H3 K27M-mutant Glioma	NCT03295396	2	Overall response rate	2023-09-30 active	95
Oral ONC201 in Recurrent GBM, H3 K27M Glioma, and Midline Glioma	NCT02525692	2	Progression-free survival as assessed by using RANO-HGG criteria	2023–12 active	89
ONC201 in Recurrent or Metastatic Type II Endometrial Cancer Endometrial Cancer	NCT03485729	2	Progression-free survival	2022-12-31 active	30
ONC201 for the Treatment of Newly Diagnosed H3 K27M-mutant Diffuse Glioma Following Completion of Radiotherapy: A Randomized, Double-Blind, Placebo-Controlled, Multicenter Study	NCT05580562	3	Overall survival; progression free survival as assessed by using RANO-HGG criteria	2026–08	450
Phase II Study of ONC201 Plus Weekly Paclitaxel in Patients with Platinum-Resistant Refractory or Recurrent Epithelial Ovarian, Fallopian Tube, or Primary Peritoneal Cancer	NCT04055649	2	Incidence of treatment related adverse events; incidence of dose limiting toxicities	2026-04-28	62
			objective response rate		
			progression free survival		
Phase I/II Study of Oral ONC201 in Patients with Relapsed or Refractory Acute Leukemias and High-Risk Myelodysplastic Syndromes	NCT02392572	1 and 2	Maximum tolerated dose (Phase I); objective response (Phase II)	2024-11-30	120
Phase I Study of Oral ONC206 in Recurrent and Rare Primary Central Nervous System Neoplasms	NCT04541082	1	Maximum tolerated dose of single-agent, oral; number of participants who experienced dose-limiting toxicities	2025–02	102
ONC206 for Treatment of Newly Diagnosed, Recurrent Diffuse Midline Gliomas, and Other Recurrent Malignant CNS Tumors (PNOC023)	NCT04732065	1	Proportion of participants with dose-limiting toxicities; maximum tolerated dose	2027-12-31	256

In a recent review article, Karp and Lyakhovich outlined antibiotics that, by inducing mitochondrial dysfunction, hinder OXPHOS and the rate of oxygen consumption, reduce ATP and ΔΨm levels, and increase ROS (Karp and Lyakhovich, 2022). Antibiotics act on the prokaryotic ribosomal complex by binding to the bacterial 30S ribosomal subunit, thus preventing association with aminoacyl transfer RNAs (tRNAs) and counteract translation with a consequent bacteriostatic effect (Luger et al., 2018). Translation of mitochondria-encoded proteins occurs within the mitoribosome organelle and produces 13 proteins that are components of respiratory complexes (Luger et al., 2018). The evolutionary conserved link between mitochondria and bacteria supports the use of these drugs to target CSC metabolism (Luger et al., 2018; Karp and Lyakhovich, 2022). Bedaquiline is an anti-microbial agent that is approved by the FDA for the treatment of resistant tuberculosis. It significantly blocks the expansion CSCs generated by breast cancer MCF7 cell line, as determined by reduced expression of CD44 and ALDH1, under anchorage-independent growth conditions and the mammosphere assay (Fiorillo et al., 2016a). Several preclinical studies support efficacy of doxycycline against CSCs (Lamb et al., 2015; Yang et al., 2015; Liu et al., 2022). Lamb et al. found that doxycycline was effective against tumor-sphere formation across different cancer types including breast, ovarian, prostate, lung, pancreatic cancers, melanoma, and glioblastoma (Lamb et al., 2015). In early breast cancer patients, Scatena et al. conducted a clinical pilot study with doxycycline finding a significant decrease in cancer tissues of two CSC markers, namely, CD44 and ALDH1 (Scatena et al., 2018). Yang et al. show that doxycycline severely affected colony formation and viability of human cervical carcinoma stem cells (He-La CSCs), decreased expression of SOX-2 and surface markers CD133 and CD49f. Moreover, upon injection into NOD-SCID mice the doxycycline pretreated HeLa-CSCs had drastically reduced capacity of tumor growth (Yang et al., 2015). Liu et al. showed that the drug significantly inhibited the CSC-like properties of pancreatic cancer cells, namely, mammosphere formation and CD133 expression (Liu et al., 2022). Treatment of Panc-1 with doxycycline significantly enhanced the effect of chemotherapy drugs (i.e., cisplatin, oxaliplatin, 5-FU, sorafenib, and gemcitabine) in comparison with the results obtained when only chemotherapy drugs were used. Among the antibiotics with preclinical evidence of efficacy to suppress CSCs, for which we refer *ad hoc* review articles (Karp and Lyakhovich, 2022; Garimella et al., 2023), doxycycline, a tetracycline derivative is the most investigated in clinical trials. Clinical trials investigating the drug alone or in combination with standard therapy involve varied tumors, among which: pancreatic cancer (phase 2, NCT02775695); pleural neoplasm (observational, NCT03465774; interventional, NCT02583282; phase 2, NCT01411202); cutaneous T-cell lymphoma (phase 2, NCT02341209); advanced melanoma, in association with temozolomide and ipilimumab (phase 1, NCT01590082); relapsed NHL (phase 2, NCT02086591); bone metastatic breast cancer, in association with bisphosphonates (NCT01847976); in localized breast cancer and uterine cancer (phase2, NCT02874430) or head and neck cancer (phase 2, NCT03076281), in association with metformin. Tigecycline, a glycylcycline designed to overcome tetracycline resistance was shown to interfere with the generation of CSCs (LGR5⁺CD44⁺) in a colon adenocarcinoma murine model (Ruiz-Malagón et al., 2023). Moreover, tigecycline impacted tumorsphere formation in a number of cancer cell lines, including ER (−) breast, ovarian, lung, prostate, and pancreatic cancers and melanoma (Lamb et al., 2015). Currently, tigecycline is investigated in acute and chronic myeloid leukemia (phase 1, NCT01332786; observational, NCT02883036). The macrolide azithromycin exerted a very significant inhibitory effect on mammosphere formation when combined with doxycycline (Fiorillo et al., 2019). It is investigated in Familial Adenomatous Polyposis (FAP) carrying premature nonsense mutations (phase 4, NCT04454151). Table 3 resumes active or recently completed clinical trials investigating outcomes of antibiotics in refractory tumors.

**TABLE 3:** Clinical trials investigating the outcomes of tumor treatments with antibiotics.

Title	ClinicalTrials. gov ID	Phase	Primary outcome measure	Study completion, actual/estimated	Enrollment, actual/estimated
Efficacy of Doxycycline on Metakaryote Cell Death in Patients with Resectable Pancreatic Cancer	NCT02775695	2	The number of dead/dying metakaryotes per 1 g of tissue. and the plasma drug concentrations	2022–05	12
Indwelling Pleural Catheters with or without Doxycycline in Treating Patients With Malignant Pleural Effusions	NCT03465774	Observational	Time to pleural catheter removal; recurrence of effusion; quality-adjusted survival; dyspnea	2025–04	208
Metformin Hydrochloride and Doxycycline in Treating Patients with Localized Breast or Uterine Cancer	NCT02874430	2	To percentage of cells that express caveolin-1, MCT1, MCT4 and TOMM20 at baseline and after treatment; safety and tolerability	2023–06	27
Azithromycin Treatment for Readthrough of APC Gene Stop Codon Mutations in Familial Adenomatous Polyposis (FAP)	NCT04454151	4	Evaluation of changes in number and size of adenomas measured by upper endoscopy	2022–04	10

CPI-613 (devimistat) is a nonredox active lipoate analog developed by Cornerstone Pharmaceuticals. CPI-613 mimics the cofactor of the E2 catalytic subunit of pyruvate dehydrogenase and ketoglutarate dehydrogenase (Stuart et al., 2014), inhibiting the enzymatic activity of these complexes operating on the TCA cycle (Stuart et al., 2014) and impairs ATP synthesis (Anderson et al., 2022). Also, TCA cycle inhibition leads to increased mitochondrial turnover due to mitophagy (Anderson et al., 2022).

**FIGURE 4:**
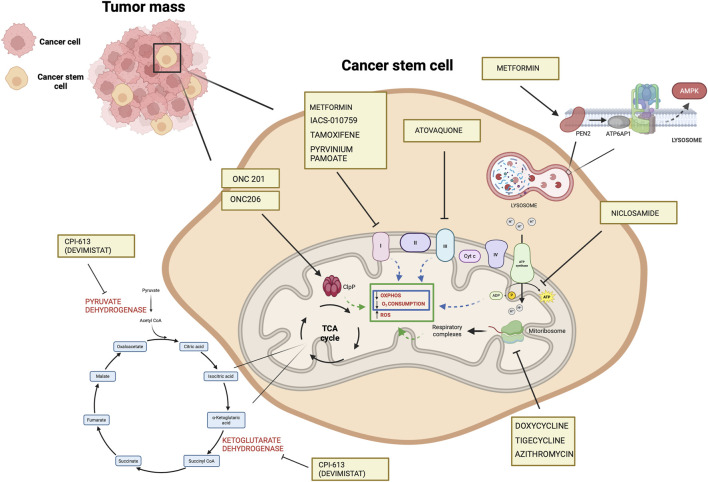
Mechanisms of the mitochondrial drugs used in clinical trials that have been shown to target CSCs in preclinical setting (https://www.clinicaltrials.gov). The illustration was started from scratch, created with BioRender.com original design.

In ovarian cancer, CPI-613 treatment was found to negatively impact CSC-rich spheres and resulted in a decrease in tumorigenicity *in vivo*. Moreover, CPI-613 treatment induced a decrease in CD133^+^ and CD117^+^ cell frequency *in vitro* and *in vivo* (Bellio et al., 2019). In early clinical trials in pancreatic cancer patients, devimistat produced impressive response rates (Alistar et al., 2017) leading to a phase 3 clinical trial (Philip et al., 2019). Moreover, in preclinical models, devimistat sensitized AML cells to chemotherapy and decreased mitochondrial respiration, leading to a phase I study in relapsed and refractory AML patients (Pardee et al., 2018). However, devimistat did not improve overall survival in the multi-center phase 3 randomized clinical trial (NCT03504423) where 528 patients with metastatic pancreatic adenocarcinoma were randomized to receive either devimistat in combination with modified Folfirinox or Folfirinox (Rafael Pharmaceuticals, Inc., 2021). Similarly, the phase 3 study ARMADA 2000 (NCT03504410) was not completed due to a lack of efficacy in patients with relapsed or refractory AML (Anderson et al., 2022). Despite preliminary unsuccessful results, investigations in the clinics continue with the aim of assessing with more precision devimistat capabilities against difficult-to-treat tumors and defining the best condition for devimistat use. Table 4 lists active clinical trials evaluating this drug in the treatment of advanced/refractory tumors. Figure 4 illustrates the mechanism of action of anti-mitochondrial drugs used in clinical trials.

**TABLE 4:** Clinical trials investigating the outcomes of the treatments of refractory tumors with CPI-613 (Devimistat).

Title	ClinicalTrials. gov ID	Phase	Primary outcome measure	Study completion, actual/estimated	Enrollment, actual/estimated
A Study of CPI-613 for Patients with Relapsed or Refractory Burkitt Lymphoma/​Leukemia or High-Grade B-Cell Lymphoma with High-Risk Translocations	NCT03793140	2	Overall response rate will be defined as rate of complete response + partial response + minor response + stable disease as determined as per the criteria for response assessment in lymphoma (RECIL)	2024–12	24
Open Label Phase I/​II Clinical Trial to Evaluate CPI-613 in Patients with Advanced Malignancies	NCT00741403	1	To evaluate the safety, tolerability, maximum tolerated dose, and efficacy pharmacokinetics of CPI-613 given twice weekly for three consecutive weeks in cancer patients	2016–12 actual	39
Phase 2 Safety, Tolerability and Efficacy Study of CPI-613 in Cancer Patients	NCT01832857	2	Overall survival	2016–12 actual	7
CPI-613 (Devimistat) in Combination with Chemoradiation in Patients with Pancreatic Adenocarcinoma	NCT05325281	1	Maximum tolerated dose will be determined by testing increasing doses of CPI-613, starting from 500 mg/m2 and up to 1,500 mg/m2, on dose escalation cohorts of three patients in combination with Gem-RT therapy	2027–08	24
A Study of CPI-613 for Patients with Relapsed or Refractory Burkitt Lymphoma/​Leukemia or High-Grade B-Cell Lymphoma with High-Risk Translocations	NCT03793140	2	Overall response rate will be defined as rate of complete response + partial response + minor response + stable disease as determined as per the criteria for response assessment in lymphoma (RECIL)	2024–12	24
CPI-613 in Combination with Modified FOLFIRINOX in Locally Advanced Pancreatic Cancer	NCT03699319	1 and 2	Overall survival Maximum tolerated dose of CPI-613 in combination with mFOLFIRINOX in the added small cohort of participants with higher doses of CPI-613 developed to redefine maximum tolerated dose	2024–10	49
CPI-613 Given with Metformin in Patients with Relapsed or Refractory Acute Myeloid Leukemia (AML)	NCT05854966	2	Number of participants to receive at least one cycle of maintenance therapy -feasibility	2025–09	17
CPI-613 in Combination with Bendamustine in Patients with Relapsed/​Refractory T-Cell Non-Hodgkin Lymphoma	NCT04217317	2	Number of participants to successfully complete therapy regimen	2025–06	12
Gemcitabine and Cisplatin with or Without CPI-613 as First Line Therapy for Patients with Advanced Unresectable Biliary Tract Cancer (BilT-04)	NCT04203160	1 and 2	Maximum tolerated dose	2025–06	78
			Overall response rate according to the RECIST criteria		
CPI-613 (Devimistat) in Combination with Hydroxychloroquine and 5-fluorouracil or Gemcitabine in Treating Patients with Advanced Chemorefractory Solid Tumors	NCT05733000	2	Overall response rate according to the RECIST criteria	2030-03-04	94

### OXPHOS-targeted drugs affect TAMs

Several studies suggest that the effects of pharmacological agents inhibiting mitochondrial metabolism, well reported for bulk tumor cells and cancer stem cells, extend beyond tumor cells and apply also to TAMs, which can contribute to their efficacy. Metformin has the potential to shift the balance of TAMs from an immunosuppressive M2 phenotype to an antitumor M1 phenotype (Wu et al., 2022; Abdelmoneim et al., 2023). A plethora of studies on different cancer models report its efficacy against TAMs (Liu et al., 2018; Wang et al., 2020; Munoz et al., 2021; Wei et al., 2021; Kang et al., 2022; Taylor et al., 2022; Cao et al., 2023). In mice-bearing prostate tumors, metformin remarkably suppressed the infiltration of TAMs mechanistically by inhibiting the cyclooxygenase-COX-2/prostaglandin-PGE2 axis in tumors. The reduction of TAMs following administration of metformin was responsible for the suppression of tumor growth and metastasis (Liu et al., 2018). Evaluating matched pre- and post-treatment tumor specimens from esophageal cancer patients in a phase II clinical trial of low-dose metformin treatment found significant changes in the TME. Precisely, metformin produced a decrease in tumor-promoting CD163^+^ macrophages and an increase in tumor-suppressive CD11c^+^ macrophages, in CD8^+^ cytotoxic T lymphocytes and CD20^+^ B lymphocytes. Also, metformin augmented macrophage-mediated phagocytosis of esophageal cancer cells *in vitro*. Similar results of TME reprogramming were obtained with short-term metformin treatment of an esophagus cancer mouse model together with inhibition of tumor growth. (Wang et al., 2020). Employing microparticles loading metformin, Wei et al. showed their efficacy in repolarizing M2-like TAMs to into M1-like phenotype and remodeling TME by increasing the recruitment of CD8^+^ T cells into tumor tissues and decreasing immunosuppressive infiltration of myeloid-derived suppressor cells and regulatory T cells (Wei et al., 2021). Metformin combined with a tumor vaccine significantly increased the expression of M1 markers CD86 and MHC-II in TME, reduced tumor growth and inhibited lung metastasis in select tumor models (Munoz et al., 2021). A study on epithelial ovarian cancer patients showed that metformin combined with platinum, in comparison with platinum alone, significantly reduced CD68^+^ macrophages and cancer-associated MSCs in TME of 38 cancer samples (Taylor et al., 2022). Two studies of colorectal cancer TME showed that metformin decreases CD206^+^ and CD163^+^ M2 macrophages in an AMPK-dependent manner (Kang et al., 2022) and promotes the polarization of TAMs to M1 through inhibition of HIF-1α and mTOR signal (Cao et al., 2023). A role for tamoxifen in TAM reprogramming to the M1 phenotype has been demonstrated in pituitary adenoma, resulting in inhibition of the migration of cancer cells. Mechanistically, such reprogramming was mediated by STAT6 inactivation and inhibition of the macrophage-specific protein tyrosine phosphatase SHP (Lv et al., 2022). Tamoxifen in combination with clodronate caused TAM depletion in castration-resistant ER-positive subtype of prostate cancer tumors (Semenas et al., 2021). It should be however noted that an expansion of an M2 population in the TME connoted tamoxifen resistance in the postmenopausal breast cancer (Xuan et al., 2014). Atovaquone, used within a stabilizer drug delivery platform composed by protoporphyrin IX nanoparticles, induced M2-type TAMs polarization toward M1-type TAMs, transforming “cold tumor” into “hot tumor” and synergized with anti-PD-L1 immunotherapy in a murine model of colon carcinoma (Feng et al., 2023). ONC201 affects macrophage immunometabolism and leads to a pro-inflammatory TME in glioblastoma (Geiß et al., 2021). Doxycycline inhibits M2-type polarization of human and bone marrow-derived mouse macrophages in a dose-dependent manner and *in vivo* M2-mediated neovascularization in a laser injury model of choroidal neovascularization (He and Marneros, 2014). In pulmonary metastases of osteosarcoma, doxycycline affects macrophage polarization by skewing the tumor induced M2-like TAMs to anti-tumor M1-like subsets, through this mechanism it prevents the progress of pulmonary micro-metastases to macro-metastases at early-stage disease. (Hadjimichael et al., 2022). Table 5 illustrates studies investigating the effects of OXPHOS-targeted drugs on TAMs.

**TABLE 5:** Effects of OXPHOS-targeted drugs on TAMs.

Treatment	Effect on TAMs	Mechanism	Cancer Type/Model	Reference
Metformin	Shifts TAMs from M2 to M1 phenotype	COX-2/PGE2 axis inhibition	Prostate tumors	Liu et al. (2018)
		Reprogrammed TAMs ↓CD163+, ↑CD11c+	Esophageal	Wang et al. (2020)
		Increased recruitment of CD8^+^ T cells	Hepatocellular carcinoma	Wei et al. (2021)
		Reprogrammed TAMs ↑CD86+	Breast cancer	Munoz et al. (2021)
			Lung carcinoma	
			Oral Squamous Cell Carcinoma	
		Reprogrammed TAMs ↓CD68+	Epithelial ovarian cancer	Taylor et al. (2022)
		AMPK-dependent ↓CD206+	Colorectal cancer	Kang et al. (2022)
		HIF-1α and mTOR inhibition	Colorectal cancer	Cao et al. (2023)
Tamoxifen	Shifts TAMs from M2 to M1 phenotype	STAT6 inactivation, SHP inhibition	Pituitary Adenoma	Lv et al. (2022)
	TAMs depletion	PIP5K1α/AKT and MMP9/VEGF axis inhibition	Prostate (ER-positive subtype)	Semenas et al. (2021)
	Expansion of M2 population	↑CD163+ macrophages infiltration	Postmenopausal Breast	Xuan et al. (2014)
Atovaquone	Shifts TAMs from M2 to M1 phenotype	Reprogrammed TAMs ↓CD206+, ↑CD11c+	Colon carcinoma	Feng et al. (2023)
ONC201	Inhibition of OXPHOS	Activation of ClpP	Glioblastoma	Geiß et al. (2021)
Doxycycline	Inhibits M2-type polarization of macrophages	IL-4-induced luciferase activity and MRC1 inhibition	Choroidal neovascularization	He L et al. (2014)
	Shifts TAMs from M2 to M1 phenotype	↓MMPs, ↓VEGF	Pulmonary metastases of osteosarcoma	Hadjimichael et al. (2022)

### Non-pharmacological disruption of energy metabolism

Studies conducted on triple-negative breast cancer (TNBC) suggest that starvation could have a therapeutic value in cancer. Specifically, Salvadori et al., showed that fasting mimicking (FMD) starvation produced a significant impairment of CSCs (CD44^+^CD24^−^) compared with non-CSC in TNBC murine model and decreased mammosphere generation and volume. FMD delayed tumor progression in a syngeneic TNBC mouse model. Moreover, combining FMD cycles with PI3K/AKT/mTOR inhibitors resulted in long-term animal survival and reduced the treatment-induced side effects (Salvadori et al., 2021). The authors suggest that FMD-induced depletion of TNBC CSCs when tumors are in a less advanced stage could enormously enhance the efficacy of subsequent treatments targeting both CSCs (such as the FMD) and more differentiated cancer cells (such as PI3K/AKT/mTORC1 inhibitors) in late-stage cancers (Salvadori et al., 2021).

Similarly, Pateras et al. showed that short-term starvation increased sensitivity to DNA-damaging chemotherapeutic agents (doxorubicin or cisplatin) and inhibited oxidative stress-induced DNA damage repair in TBNC cells. Mechanistically, the combination of STS and chemotherapy-induced an increase of ROS production in such cancer cells through a collapse of mitochondrial respiration and an altered ATP production. In contrast, in normal, non-transformed cells, this combination has a protective effect (Pateras et al., 2023). The reasons for the differential response of normal *versus* cancer cells to dietary restriction remain unknown. More insights into starvation-induced mechanisms may lead to safe and effective anti-cancer treatments and help to overcome the chemotherapy resistance of cancer. Future *ad hoc* designed clinical trials are needed to assess dietary recommendations as an adjunct to chemotherapy for TNBC treatment and to confirm the efficacy of the combined approach.

### Concluding remarks

In various tumors, CSCs and their supporting macrophages have been shown to be highly dependent on mitochondrial function and OXPHOS metabolism. Such a metabolic dependency of CSCs has stimulated modern chemotherapy targeting mitochondria/OXPHOS for cancer cure. To date, numerous clinical trials are underway across a wide range of advanced, resistant, and refractory tumors with a wide range of anti-mitochondrial and anti-metabolic agents. Numerous further agents are the subject of preclinical investigations linking laboratory drug discovery to the initiation of human clinical trials. However, the clinical use of pharmacological agents targeting such metabolic vulnerabilities of CSCs presents numerous challenges. There are issues related to the toxicity of antimitochondrial drugs; also, results so far obtained with clinical trials are sometimes vague or unflattering. More studies are needed to codify and quantify drug effects on healthy cells and find a therapeutic window and valuable tools that assist personalized therapies for precise administration indication. Despite these issues, awareness of the metabolic plasticity of CSCs supports perseverance in the anti-mitochondrial therapeutic approach. An increasing number of investigations of anti-mitochondrial medications in clinical trials are underway to hinder hard-to-treat tumors. The precise definitions of the therapeutic window and dose of the drug, mode of administration, optimization strategies for selective delivery to tumor cells, and combination with distinct targeted agents are currently being investigated in an attempt to guarantee a safety profile and at the same time undermine CSCs and their selection advantage that causes relapse. Moreover, the implementation of *ad hoc* phase 1 and 2 studies could accelerate the combined use of drugs potentially active against CSCs with those of standard cancer protocols, thus improving helpful information to adopt the use of such combination as the first line of intervention against tumors with a high frequency of recurrence.

## Limitation

We have not described the influence of all the components of the niches that vary between solid tumor, and leukemia, as well as primary and metastatic tumor. Still, we focused on TAMs because the study of the niche components would have opened up very broad scenarios that deserve to be treated and explored in depth in a separate article. Furthermore, the studies we have presented often extend to the concept of stem cell-like cells and refer to specific tumors and experimental contexts that are not generalizable to different CSCs from all kinds of tumors.
